# Home range size, habitat selection and roost use by the whiskered bat (*Myotis mystacinus*) in human-dominated montane landscapes

**DOI:** 10.1371/journal.pone.0237243

**Published:** 2020-10-09

**Authors:** Korneliusz Kurek, Olga Gewartowska, Katarzyna Tołkacz, Bogumiła Jędrzejewska, Robert W. Mysłajek

**Affiliations:** 1 Department of Ecology, Institute of Functional Biology and Ecology, Faculty of Biology, Biological and Chemical Research Centre, University of Warsaw, Warszawa, Poland; 2 Department of Antarctic Biology, Institute of Biochemistry and Biophysics, Polish Academy of Sciences, Warszawa, Poland; 3 Mammal Research Institute, Polish Academy of Sciences, Białowieża, Poland; Wildlife Conservation Society Canada, CANADA

## Abstract

Our understanding of animal adaptations to human pressure is limited by the focus on rare taxa, despite that common species are more significant in shaping structure, function and service provision of ecosystems. Thus better understanding of their ecology and behavioural adjustments is central for drafting conservation actions. In this study, we used radio-telemetry on 21 individuals (10 females, 11 males) to provide data on spatial ecology, habitat selection and use of roosts of one of the commonest species, the whiskered bat (*Myotis mystacinus*), inhabiting the Carpathian Mountains (southern Poland). We tested, whether this species prefers natural over human-modified landscapes to seek prey and roosts. Mean home range size of the whiskered bat in the Carpathian Mountains was 26.3 ha (SE ± 3.2, Local Convex Hull) and 110 ha (SE ± 22.1, Minimum Convex Polygon with all locations), and included between one and three patches, among which bats moved along linear environmental features, such as scrubby banks of streams or lines of trees. During foraging whiskered bats selected small woodlands within agricultural landscapes, avoided large mountain forests and open areas, and used built-up areas proportionally to their availability. Whiskered bats occupied roosts located mainly in buildings (>97%), at an average altitude of 547.9 m above sea level (SE ± 8.3). Roosts were used for 5.4 days, on average. Our study shows that whiskered bats adapted well to the mosaic of semi-natural and anthropogenic habitats. It highlights the importance of buildings serving as roosts and small woodlands used as foraging areas in human-dominated montane landscapes.

## Introduction

Deterioration and loss of habitats through deforestation, intensification of agriculture, urbanization, development of transport infrastructure, and wind farms threaten bat populations worldwide [1, 2]. By contrast, the most widespread and abundant species of bats exhibit various behavioural adjustments enabling them to thrive even in heavily urbanized areas [3–5]. Unfortunately, our understanding of species adaptations to human pressure is limited by the fact that researchers often focus on rare taxa, which are by default classified as being at risk. It also has important drawbacks, e.g. bias towards species with small populations, limited ranges or narrow habitat preferences. Nonetheless, common species are more influential in shaping structure, function and service provision of ecosystems. Therefore knowledge about their ecology and adaptations is crucial for planning conservation actions [6, 7].

The whiskered bat (*Myotis mystacinus*) (Kuhl, 1817) is one of the most common bats in Europe [8]. It is a small (4–7 g; [8]), sedentary [9] and insectivorous species [10–12] that forages mainly in cluttered (i.e. obstacle dense) habitats [13, 14]. Some studies have suggested its relationship with open areas and vegetation along watercourses [15], while others have pointed to forests [16, 17]. Whiskered bats have been found hibernating in caves with a wide range of microclimate conditions [18], while their breeding colonies were set up mainly in buildings [17, 19, 20].

The whiskered bat is abundant in the Western Carpathian Mountains [21–23]. In this montane range environmental features and human pressure varies with altitude creating convenient conditions for studies on the adaptations of wild animals to an urban–rural–forest landscape gradient [24, 25]. We used radio-telemetry to record data on spatial ecology, habitat selection and use of roosts of the whiskered bat in a densely populated fragment of the Polish Carpathians. We attempted to test whether this species prefers more natural (i.e. forests) over human-modified (i.e. urban and agricultural) landscapes to seek prey and roost.

## Methods

### Study area

The study area (70 km^2^) was situated in the western-most part of the Polish Carpathian Mountains (49°40'00.9"N 19°04'35.2"E), at the transition zone between Żywiec Basin and Silesian Beskid Mountains. The altitude within the area varies between 250 and 1,257 m above sea level (hereafter asl). Most of the local caves are characterized by high instability and narrow, labyrinthine systems of cavities with numerous depressions and slits [26]. They have a stable microclimate with high humidity and a temperature ranging from 2 to 8°C in winter. Temperature, precipitation, and snow cover is very variable and depends on the altitude (S1 Table). Historically, the study area was covered almost entirely with forests. Rich broadleaved woods with oak (*Quercus* ssp.), lime (*Tilia* ssp.), hornbeam (*Carpinus betulus*) dominated the foothills, beech (*Fagus sylvatica*), sycamore (*Acer pseudoplatanus*) and fir (*Abies alba*) prevailed in the lower montane zone, while the upper montane zone was covered mainly by Norway spruce (*Picea abies*). Centuries of deforestation, development of agriculture and human settlements changed these landscapes. Nowadays, the foothills are mainly covered with arable fields, pastures, meadows, villages and towns, while small woods remain mainly along watercourses and summits. Although both lower and upper montane zones remain forested, species composition of forests has changed towards the domination of managed stands of Norway spruce and beech trees, with an admixture of fir [27, 28]. Human population density is 147 people/km^2^ [29]. Smaller villages are expanded along roads and smoothly merge into each other. In the course of the twentieth century, traditional wooden cottages with gable roofs were gradually replaced with concrete brick houses covered with flat tiles or tar paper.

### Telemetry

Between June and September 2009–2011, we captured 33 whiskered bats (15 females, 18 males) with mist-nets (S2 Table) along water courses and near buildings occupied by the breeding colonies of bats, as well as near caves. All individuals were subjected to genetic analysis based on mtDNA sequence, which confirmed the accuracy of the species identification [30]. We marked captured bats with metal bands (Porzana, UK). Then, we equipped them with radio-telemetric transmitters weighing 0.39 g (BD-2N, Holohil, Canada) or 0.30 g (PIP4, Biotrack, UK), which corresponds to 4–7.8% of their body weight. Transmitters were placed using surgical glue (Torbot, USA) on a shaved area of the back, between the shoulders. Two individuals were caught again, a year after the completion of radio telemetry measurements: in both cases, the transmitter had been dropped and the shaved fur was fully replaced. Due to the wet weather one individual spent most of its time roosting, and another 11 individuals (including all marked at the time of swarming) left the study area shortly after the mounting of transmitters (later one was found about 10 km from the original site of capture). Therefore, for the analysis of home-range size and habitat preferences we used data obtained from 21 individuals (10 females, 11 males) (S2 Table).

Marked individuals were tracked with a directional antenna (type RA-14, Telonics, USA or YAGI-AY/C, Titley Electronics, Australia) and radio VHF receiver (type VR-500, Yaesu, Japan). The positions of the radio tagged bats were determined by triangulation [31] with 2–3 fixes. We attempted to obtain bat positions at least every 15 minutes between dusk and dawn. The location error, estimated experimentally by triangulation of the transmitter with known locations, averaged 94 ± 4 m (mean ± SE).

### Ethical statement

Capturing and tagging of bats was conducted under the Regional Directorate for Environmental Protection in Katowice (Poland) permit and all procedures on animals were reviewed and approved by the 1^st^ Local Ethical Commission for Animal Experiments in Warsaw (Poland).

### Calculation of home-range size

Based on the data obtained from radio telemetric measurements, home range sizes were determined, as well as the daily activity and habitat preferences of bats, both in relation to feeding sites and daytime hiding places. To calculate the home ranges we applied a local convex hull (hereafter LoCoH) nonparametric kernel method [32, 33] with fixed number of points *k* = 10 (*k* is the number of the nearest points used to calculate a single area). The calculations were made through the site http://locoh.cnr.berkeley.edu allowing for analysis of on-line data collected, as well as using the R statistical software [34]. Furthermore, to allow comparison of our results with other studies, we also calculated home range size using a Minimum Convex Polygon with 100% of locations (hereafter MCP). We performed MCP calculations in R statistical software with the adehabitatHR package [34, 35].

### Assessment of habitat preferences

We determined habitat preferences of bats based on Ivlev’s selectivity index modified by Jacobs [36] according to the formula:

$$D=\frac{r-p}{r+p-2rp}$$

where: *r* = proportion of given habitat within home range or proportion of time spend by the bat within it and *p* = proportion of given habitat within the study area or theoretical proportion of time spend by the bat in this habitat calculated from the proportion of the habitat within the study area.

Ivlev’s index ranges from -1 (total avoidance) through 0 (no selection) to 1 (strong preference). Environmental parameters around the roosts occupied by bats were compared to the parameters of random points within the home ranges generated with GIS software (MapInfo Professional, Pitney Bowes Inc., USA). The following environmental parameters were taken into account: (1) altitude, and the minimum distances from (2) the nearest road, (3) water sources, (4) small woodlands, (5) open area, and (6) dense forest. For comparisons we used randomly selected points within the home ranges of bats.

## Results

### Home ranges

Bats selected for the home range analysis (i.e. 10 females and 11 males with suitable number of locations (S2 Table)) were tracked between 5 and 14 nights (9.1 ± 0.6, mean ± SE). From 162 to 593 locations were obtained from each individual (319 ± 25.3, mean ± SE) (Table 1). There was no significant effect, however, of the number of locations on the estimated size of home range (r = -0.43, p = 0.19, n = 21). Between June and September, the average home range of the whiskered bats covered 26.3 ha (SE ± 3.2) LoCoH and 110.0 ha (SE ± 22.1) MCP. Home ranges of females and males did not differ in extent, neither for LoCoH (Mann-Whitney test, U = 44.0, p = 0.46) nor for MCP (Mann-Whitney test, U = 53.0, p = 0.92) (Table 1). Home ranges included between one and three patches (2.0 ± 0.2, mean ± SE).

**Table 1:** Home range size (LoCoH–local convex hull, MCP–minimum convex polygon, with all locations), number of foraging areas and number of roosts of whiskered bats tracked with telemetry in the Western Carpathian Mountains, 2009–2011.

ID	Duration of telemetry (nights)	Number of locations	Home range LoCoH (ha)	Home range MCP (ha)	Number of foraging patches	Number of roosts used
F01	9	339	38.2	51.0	2	1
F02	7	338	61.5	474.8	2	6
F03	5	346	23.4	96.2	2	1
F04	5	385	11.7	84.2	2	2
F05	9	323	47.2	210.1	2	1
F08	7	164	17.2	165.5	3	1
F09	13	241	19.9	38.7	3	3
F11	10	346	18.7	22.5	1	3
F12	7	162	22.7	79.3	2	3
F15	14	178	32.1	50.9	1	1
Mean±SE	8.6±1.0	282.2±27.4	29.3±4.9	127.3±42.8	2.0±0.2	2.2±0.5
M02	13	593	12.2	16.3	1	1
M03	8	359	48.6	97.0	1	3
M05	5	442	23.6	124.1	1	2
M06	11	244	24.5	106.3	3	2
M07	14	216	43.3	146.1	3	1
M08	7	223	38.5	147.2	2	3
M13	11	285	14.9	30.9	2	1
M15	9	356	10.9	76.0	2	3
M16	11	205	15.0	82.2	2	2
M17	8	410	8.8	13.2	1	1
M18	8	537	20.2	197.8	3	2
Mean±SE	9.6±0.8	351.8±40.1	23.7±4.2	94.3±17.7	1.9±0.3	1.9±0.3

See S2 Table for the detailed description of studied individuals.

The number of patches used by both sexes was similar (Mann-Whitney, U = 51.0, p = 0.79). Bats moved among patches usually along the linear environmental features (plants growing on the banks of streams, lines of trees). On nights with bad weather bats restricted their activity to a single feeding ground, and returned to the roost earlier. In three cases, the bats (adult males) spent one to four nights at the feeding ground located away from the roost used on regular basis. In such cases they spent the day roosting in the vicinity of the feeding grounds.

During the tracking period (mean 9 days) whiskered bats occupied an average of 2.1 (SE ± 0.3) roosts. Females used from 1 to 6 roosts, whereas males from 1 to 4 (Table 1), yet the difference was not significant (Mann-Whitney test, W = 54.5, p = 1.00).

### Environmental preferences

Whiskered bats spent the longest activity time in small woodlands located within agricultural lands (40.8%), compared to montane forests and open areas (27%), while in built-up areas they spent only 4% of the recorded time (Table 2).

**Table 2:** Available habitat area (% within home range area) in home-ranges of whiskered bats, and percentage of time spent by active bats in those habitats (mean±SE).

Habitat	Study area	Home ranges			Bat activity		
		Males	Females	All	Males	Females	All
Montane forest	32.8	25.6±7.0	23.8±8.0	24.8±5.2	25.1±8.5	30.0±11.8	27.4±7.0
Small woodlands	8.1	27.2±3.8	26.7±3.8	27.0±2.5	38.6±7.2	43.1±7.1	40.8±5.0
Built-up areas	3.9	5.6±1.0	8.0±2.5	6.7±1.3	4.4±1.2	3.3±1.2	3.9±0.8
Open areas	55.2	41.6±5.6	41.4±7.7	41.5±4.5	31.9±7.0	23.6±5.5	27.9±4.5

Whiskered bats favoured small woodlands within agricultural landscapes (selectivity index D = 0.56, SE ± 0.04), used built-up areas, where daytime roosts are localized, proportionally to their occurrence (D = 0.13, SE ± 0.08), and generally avoided montane forests (D = -0.22, SE ± 0.12) and open areas (D = -0.26, SE ± 0.09). Comparison of time spent by bats in the given habitat to its participation in the surface area showed that the bats used forests (D = -0.01, SE ± 0.12) and small woodlands (D = 0.10, SE ± 0.10) proportionally to their occurrence, and avoided buildings (D = -0.34, SE ± 0.08) and open areas (D = -0.36, SE ± 0.10).

Although females strongly avoided open areas and built-up areas and were more likely to forage in forests, habitat preferences of both sexes were similar. No statistically significant differences were found either in number of locations within given habitat (Student's t test: t = -0.3093; df = 16.2; p = 0.76 for forests, t = 0.0714, df = 19, 0; p = 0.94 for small woodlands; t = 0.7375, df = 18.9; p = 0.47 for build-up areas; t = 0.069, df = 16.9; p = 0.95 for open areas) or in the length of the activity (t = 0.6843; df = 13.6; p = 0.50 for forests, t = -0.0715, df = 14.5; p = 0.94 for small woodlands, t = -1.4947; df = 19.0; p = 0.15 for built-up areas; t = -1.2474; df = 17.9; p = 0.23 for open areas).

### Daytime roosts

In the course of radio telemetry, the tracked individuals were found in daytime roosts 191 times. A total of 38 roosts were found at an average altitude of 547.9 m asl (SE ± 8.3). In 97.9% of cases, the bats remained in roosts of anthropogenic origin, primarily in residential houses (23 roosts) and cottages or abandoned households (5 roosts) (S3 Table). The frequency of using a particular type of roost was not associated with their number (G test, df = 2, G = 7.0, p <0.03 for females; df = 3, G = 143.82, p <0.001 for males; df = 3, G = 82.07, p <0.001 for all individuals). The females were observed only in farm outbuildings or residential houses, while 94% of roosts used by males were of anthropogenic origin (S3 Table). Natural roosts (n = 4) located in the forest were at an average altitude of 667.2 (± SE 88.3) m asl.

Daytime roosts were located significantly closer to the montane forest (females: 430 m ± 14; males: 230 m ± 4, mean ± SE), and further away from open areas (females: 100 m ± 3; males: 60 m ± 3, mean ± SE) than the random points. There was no difference in this respect between the two sexes (Mann-Whitney U-test, U = 221, p = 0.54 for the minimum distance from the montane forest; U = 253.5, p = 0.13 for the minimum distance from the open area). Bat roosts were located at higher altitudes (533.6 m asl ± 4.2, mean ± SE) than random points (505.8 m asl ± 6.9, mean ± SE). Roosts of whiskered bats were located an average 80 m (SE ± 2) from the nearest road, 167 meters (± 2 SE) from a source of water, and 61 m (SE ± 8) from small woodlands. However, there was no significant difference between these parameters compared to random points (Mann-Whitney U test, respectively: U = 825.5, p = 0.51; U = 859, p = 0.72 and U = 1088 5, p = 0.10).

Bats spent from 1 to 11 days in one roost (mean 5.4 ± 0.7 SE). On average, they used 0.28 roosts per day (Table 3). In the course of the study 7 individuals (5 females and 2 males) never changed their daytime roost. There were no statistically significant differences between males and females in the frequency of changes made between roosts (Mann-Whitney test, U = 52.5, p = 0.89).

**Table 3:** Number of roosts and frequency of roost change by male (n = 11) and female (n = 10) whiskered bats studied with telemetry in the Western Carpathian Mountains, 2009–2011.

Parameter	Males		Females		Total
	Mean±SE	Range	Mean±SE	Range	Mean±SE
N roosts per day	0.29±0.05	0.09–0.5	0.27±0.08	0.09–1.0	0.28±0.05
N roost changes per day	0.18±0.04	0–0.5	0.21±0.08	0–0.83	0.19±0.04

## Discussion

The average home range of whiskered bats in the Western Carpathians was half as large as recorded in Ireland (MCP with all locations 110 ha and 228 ha, respectively) [17]. These differences may be due to the spatial distribution of environmental patches constituting the preferred feeding grounds for bats. In the Western Carpathians, clumps of trees were not as strongly separated as in Ireland. In the Western Carpathians, as in the Irish population, home ranges of whiskered bats overlapped, indicating a lack of territorialism in this species. In other species of bats researchers also observed smaller [37, 38] or larger [39–41] overlapping of home ranges and did not find the territorial behaviour. Contrary to the earlier reports of defending the feeding grounds in this species [39] whiskered bats ignored the presence of other individuals on their feeding grounds. Whiskered bats tracked by us showed fidelity to their feeding grounds. Such a mechanism [42] may be an important factor determining the distribution of bat home ranges [37, 38].

There is no agreement in the literature on the habitat preferences of the whiskered bat. While in some regions they mainly forage in the open areas and riparian woodlands [10], in others they are generally associated with different types of forests [15]. A radio telemetry study carried out in Ireland, on the edge of the species range, showed that whiskered bats foraged primarily in vegetation on the banks of streams and in small-sized mixed forests [17], which, however, should be treated as groves scattered over farmland, but not a compact forest complex. Our study also demonstrated that bats tended to avoid open areas (pastures, lawns and recreation areas). In the Western Carpathians, whiskered bats, while foraging, avoided open areas and preferred small woodlands, both the groves and tree clusters in fields as well as the vegetation of the banks of streams. In the dense forest bats forage only occasionally, probably taking advantage of the periodic abundance of emerging insects. Our results are similar to those obtained by Buckley [17] in Ireland, thus confirming the key role of trees for this species. As morphologically similar species *M*. *brandtii* and *M*. *alcathoe* select forest areas [8, 15, 30], different habitat preferences may be a mechanism to limit competition between those species, as in case of the soprano pipistrelle (*Pipistrellus pygmaeus*) and common pipistrelle (*P*. *pipistrellus*) [40].

In the British Isles, roosts of whiskered bats were also found primarily in houses and farm outbuildings [17, 20]. In the Western Carpathians, bats preferred roosts located in buildings near the trees. Natural roosts (in trees) were rarely found, chosen probably due to the proximity to rich feeding grounds. Buckley [17] also reported only few tree roosts of whiskered bats in Ireland.

Whiskered bats in the Carpathians used more roosts (1–6 vs. 1–4), and changed them less often (every 3.4 vs. 2.8 days), than individuals studied in Ireland [17]. One reason for the frequent roost changes might be to save energy by shortening the distance between the roost and the feeding ground [43]. This hypothesis does not explain, however, the change of roosts between neighbouring houses. This could be associated with females feeding offspring that seek comfortable place for rest. For example two of the females captured while leaving the colony where the young individuals were present, rested in another roost, returning to the colony only for about 10 minutes on consecutive nights, probably to milk their offspring. Such behaviour was observed in females of the common noctule (*Nyctalus noctula*) [8]. Another reason might be mating with males residing in the vicinity of the breeding colonies. Such a mechanism has been described for *M*. *myotis*: in course of scattering of the breeding colonies, females spent an average of four days in hiding places of individual males, where the mating occurred [44]. At the end of the summer, in bats belonging to the *Myotis* genus, the strategy of males to monopolize females changes due to the phenomenon of swarming, when copulation can also occur [45, 46].

Our study showed that whiskered bats adapts well to the mosaic of semi-natural and anthropogenic habitats. However, it also highlighted the importance of certain environmental features, such as woodlands used as foraging areas in agricultural lands. Therefore, it will be necessary for conservation planners to ensure that they take under consideration the significance of such elements for bat populations in human-dominated montane landscapes.

## Supporting information

S1 Table Characteristics of climatic zones in the Western Carpathian Mountains. (DOCX)

S2 Table Characteristics of whiskered bats studied with telemetry in the Western Carpathian Mountains, 2009–2011. (DOCX)

S3 Table Characteristics of roosts used by whiskered bats studied with telemetry in the Western Carpathian Mountains, 2009–2011. (DOCX)
