# Supplementary material for: Home range size, habitat selection and roost use by the whiskered bat (Myotis mystacinus) in human-dominated montane landscapes
Source: PLoS One. 2020 Oct 9;15(10):e0237243. doi: 10.1371/journal.pone.0237243 (PMC7546482; doi:10.1371/journal.pone.0237243)
Supplement: S1 Table — (DOCX) [file pone.0237243.s001.docx]

S1 Table. Characteristics of climatic zones in the Western Carpathian Mountains.

| Parameter | Altitude [m a.s.l.] | | | |
| --- | --- | --- | --- | --- |
|  | 250–500 | 501–750 | 751–1000 | 1001–1250 |
| Mean annual temperature (°C) | 7.4 | 6.2 | 5.3 | 3.7 |
| Annual precipitation (mm) | 1000 | 1090 | 1250 | 1360 |
| Durability of snow cover (days) | 85 | 116 | 138 | 154 |

Data after: Durło, G. 2012. Klimat Beskidu Śląskiego. Drukrol, Kraków.
