# Supplementary material for: Home range size, habitat selection and roost use by the whiskered bat (Myotis mystacinus) in human-dominated montane landscapes
Source: PLoS One. 2020 Oct 9;15(10):e0237243. doi: 10.1371/journal.pone.0237243 (PMC7546482; doi:10.1371/journal.pone.0237243)
Supplement: S2 Table — (DOCX) [file pone.0237243.s002.docx]

S2 Table. Characteristics of whiskered bats studied with telemetry in the Western Carpathian Mountainss, 2009-2011. Bold font indicates individuals excluded from the analysis due to the small number of locations.

| ID | Sex | Age | Forearm length (mm) | Body mass (g) | Day of capture | Duration of telemetry (nights) | Number of locations |
| --- | --- | --- | --- | --- | --- | --- | --- |
| F01 | F | ad | 34.8 | 6.0 | 18/07/2009 | 9 | 339 |
| F02 | F | ad | 33.5 | 7.5 | 25/08/2009 | 7 | 338 |
| F03 | F | ad | 35.1 | 5.0 | 27/07/2010 | 5 | 346 |
| F04 | F | ad | 35.1 | 6.0 | 29/07/2010 | 5 | 385 |
| F05 | F | ad | 35.4 | 5.0 | 21/08/2010 | 9 | 323 |
| **F06** | **F** | **juv** | **34.8** | **4.5** | **22/08/2010** | **1** | **15** |
| **F07** | **F** | **ad** | **34.2** | **6.5** | **07/09/2010** | **4** | **167** |
| F08 | F | ad | 35.1 | 7.0 | 10/07/2011 | 7 | 164 |
| F09 | F | ad | 34.4 | 5.0 | 20/07/2011 | 13 | 241 |
| **F10** | **F** | **ad** | **33.6** | **5.5** | **20/07/2011** | **4** | **127** |
| F11 | F | ad | 35.1 | 5.5 | 20/07/2011 | 10 | 346 |
| F12 | F | ad | 35.1 | 5.0 | 11/08/2011 | 7 | 162 |
| **F13** | **F** | **ad** | **35.8** | **5.5** | **21/08/2011** | **1** | **6** |
| **F14** | **F** | **ad** | **34.5** | **5.5** | **21/08/2011** | **1** | **5** |
| F15 | F | juv | 34.0 | 5.5 | 10/09/2011 | 14 | 178 |
| **M01** | **M** | **ad** | **34.3** | **5.0** | **03/08/2009** | **6** | **24** |
| M02 | M | ad | 34.2 | 5.0 | 17/08/2009 | 13 | 593 |
| M03 | M | ad | 34.5 | 5.0 | 23/08/2009 | 8 | 359 |
| **M04** | **M** | **ad** | **32.6** | **4.5** | **26/05/2010** | **4** | **124** |
| M05 | M | ad | 33.5 | 5.0 | 09/09/2010 | 5 | 442 |
| M06 | M | ad | 34.7 | 5.5 | 23/06/2011 | 11 | 244 |
| M07 | M | ad | 34.1 | 5.0 | 23/06/2011 | 14 | 216 |
| M08 | M | ad | 35.3 | 5.0 | 13/07/2011 | 7 | 223 |
| **M09** | **M** | **ad** | **35.1** | **5.0** | **02/08/2011** | **6** | **8** |
| **M10** | **M** | **ad** | **34.5** | **5.0** | **02/08/2011** | **4** | **104** |
| **M11** | **M** | **ad** | **34.5** | **5.0** | **02/08/2011** | **8** | **21** |
| **M12** | **M** | **ad** | **34.9** | **5.5** | **02/08/2011** | **1** | **5** |
| M13 | M | ad | 33.3 | 5.0 | 11/08/2011 | 11 | 285 |
| **M14** | **M** | **ad** | **34.7** | **4.5** | **20/08/2011** | **1** | **13** |
| M15 | M | ad | 32.3 | 5.0 | 10/09/2011 | 9 | 356 |
| M16 | M | ad | 33.4 | 5.5 | 10/09/2011 | 11 | 205 |
| M17 | M | ad | 34.0 | 6.0 | 21/09/2011 | 8 | 410 |
| M18 | M | juv | 33.1 | 5.0 | 21/09/2011 | 8 | 537 |
