# Supplementary material for: Home range size, habitat selection and roost use by the whiskered bat (Myotis mystacinus) in human-dominated montane landscapes
Source: PLoS One. 2020 Oct 9;15(10):e0237243. doi: 10.1371/journal.pone.0237243 (PMC7546482; doi:10.1371/journal.pone.0237243)
Supplement: S3 Table — (DOCX) [file pone.0237243.s003.docx]

S3 Table. Characteristics of roosts used by whiskered bats studied with telemetry in the Western Carpathian Mountains, 2009-2011.

| Sex/Habitat | Shelter type | No. of roosts | |  | No. of visits by bats | |  | Altitude (m) | |
| --- | --- | --- | --- | --- | --- | --- | --- | --- | --- |
|  |  | N | % |  | N | % |  | Mean±SE | Range |
| **Males** | | | |  | | |  | | |
| Built-up area | Inhabited house | 10 | 43.5 |  | 46 | 45.5 |  | 538.8±4.7 | 509-561 |
| Built-up area | Weekend cabin or abandoned house | 4 | 17.4 |  | 36 | 35.6 |  | 557.2±16.6 | 526-587 |
| Built-up area | Outbuilding | 3 | 13.1 |  | 13 | 12.9 |  | 523.3±2.0 | 520-527 |
| Built-up area | Bird box | 1 | 4.3 |  | 1 | 1.0 |  | 540 | - |
| Forest | Wooden wildlife feeder | 1 | 4.3 |  | 1 | 1.0 |  | 649 | - |
| Forest | Tree | 4 | 17.4 |  | 4 | 4.0 |  | 667.2±58.3 | 540-818 |
| Total | | 23 | 100 |  | 101 | 100 |  | 565.1±14.1 | 509-818 |
| **Females** | | | | | | | | | |
| Built-up area | Inhabited house | 14 | 77.8 |  | 79 | 87.8 |  | 526.1±9.9 | 423-572 |
| Built-up area | Weekend cabin or abandoned house | 2 | 11.1 |  | 9 | 10.0 |  | 534.0±3.0 | 531-537 |
| Built-up area | Outbuilding | 2 | 11.1 |  | 2 | 2.2 |  | 529.0±9.0 | 423-572 |
| Total | | 18 | 100 |  | 90 | 100 |  | 529.1±6.4 | 423-572 |
| **All individuals** | | | | | | | | | |
| Built-up area | Inhabited house | 23 | 60.6 |  | 125 | 65.4 |  | 530.2±6.4 | 423-572 |
| Built-up area | Weekend cabin or abandoned house | 5 | 13.2 |  | 45 | 23.6 |  | 547.7±10.0 | 526-587 |
| Built-up area | Outbuilding | 4 | 10.5 |  | 15 | 7.9 |  | 527.0±3.9 | 520-538 |
| Built-up area | Bird box | 1 | 2.6 |  | 1 | 0.5 |  | 540 | - |
| Forest | Wooden wildlife feeder | 1 | 2.6 |  | 1 | 0.5 |  | 649 | - |
| Forest | Tree | 4 | 10.5 |  | 4 | 2.1 |  | 667.2±58.3 | 540-818 |
| Total | | 38 | 100 |  | 191 | 100 |  | 547.9±8.3 | 423-818 |
